# Visualization of c-di-GMP in multicellular *Dictyostelium* stages

**DOI:** 10.3389/fcell.2023.1237778

**Published:** 2023-07-20

**Authors:** Hayato Ide, Yukihisa Hayashida, Yusuke V. Morimoto

**Affiliations:** ^1^ Graduate School of Computer Science and Systems Engineering, Kyushu Institute of Technology, Fukuoka, Japan; ^2^ Department of Physics and Information Technology, Faculty of Computer Science and Systems Engineering, Kyushu Institute of Technology, Fukuoka, Japan; ^3^ Japan Science and Technology Agency, PRESTO, Kawaguchi, Japan

**Keywords:** c-di-GMP, signal transduction, fluorescence microscopy, cell differentiation, *Dictyostelium*

## Abstract

The bacterial signaling molecule cyclic diguanosine monophosphate (c-di-GMP) is only synthesized and utilized by the cellular slime mold *Dictyostelium discoideum* among eukaryotes. *Dictyostelium* cells undergo a transition from a unicellular to a multicellular state, ultimately forming a stalk and spores. While *Dictyostelium* is known to employ c-di-GMP to induce differentiation into stalk cells, there have been no reports of direct observation of c-di-GMP using fluorescent probes. In this study, we used a fluorescent probe used in bacteria to visualize its localization within *Dictyostelium* multicellular bodies. Cytosolic c-di-GMP concentrations were significantly higher at the tip of the multicellular body during stalk formation.

## 1 Introduction

Cyclic diguanylate monophosphate (c-di-GMP) is a critical signaling molecule involved in diverse cellular processes including biofilm formation, motility, virulence factor production, and cell-cycle progression in prokaryotes (Romling et al., 2013; Jenal et al., 2017; Cheang et al., 2019; Homma and Kojima, 2022). In bacteria, intracellular c-di-GMP levels are tightly regulated to control the transition between the motile planktonic and the sessile biofilm states (Krasteva et al., 2012). Whereas c-di-GMP is a major signaling molecule in prokaryotes, it is rarely utilized in eukaryotes; it is an external stimulus in the innate immune response acting through the cyclic GMP–AMP synthase (cGAS)–stimulator of interferon genes (STING) pathway (Burdette et al., 2011).

On the other hand, the cellular slime mold *Dictyostelium discoideum* is the only eukaryote known to synthesize c-di-GMP and use it as an intercellular signal (Chen and Schaap, 2012; Kin and Schaap, 2021). It normally grows in a unicellular state and feeds on surrounding bacteria. However, when starved, its single cells aggregate by chemotaxis toward cAMP signals to form a multicellular body consisting of 10^4^ to 10^5^ cells. The anterior and posterior regions of this body (also called a slug) differentiate into prestalk and prespore cells, respectively; after a short migration, the slug go through a period of a culminant, eventually differentiates into a fruiting body composed of a stalk and spores (Chisholm and Firtel, 2004; Weijer, 2004; Loomis, 2015). During this process, c-di-GMP is produced by DgcA synthase, localized to the anterior region of the multicellular body. It contributes significantly to the differentiation of stalk cells by acting on adenylate cyclase A (AcaA) to stimulate the synthesis of cAMP, which in turn activates PKA and induces the expression of stalk-specific genes. While DgcA is widely expressed in the prestalk region, AcaA is expressed only in the tip region (Chen et al., 2017). It is unclear what mechanisms are responsible for this difference in spatial distribution.

*D. discoideum* is a model organism for the study of signal transduction, and sensitive fluorescence measurements of second messengers, including Ca^2+^, cAMP, and inositol phospholipids, have been reported (Arai et al., 2010; Gregor et al., 2010; Horikawa et al., 2010; Fukushima et al., 2019; Hashimura et al., 2019; Banerjee et al., 2022; Hashimura et al., 2022). However, c-di-GMP has never been observed using fluorescence techniques in *Dictyostelium*.

Several probes have been developed to detect intracellular c-di-GMP in living bacteria (Christen et al., 2010; Ho et al., 2013; Dippel et al., 2018; Halte et al., 2022). YcgR, a PilZ domain-containing protein, undergoes a conformational change upon c-di-GMP binding, functioning as a brake on the bacterial flagellar motor (Paul et al., 2010). A probe that changes FRET efficiency in a c-di-GMP concentration-dependent manner has been constructed by fusing yellow fluorescent protein (YFP) and cyan fluorescent protein (CFP) to the N- and C-termini of YcgR from *Salmonella enterica* serovar Typhimurium, respectively, to visualize asymmetric intracellular concentrations during cell divisions of *Caulobacter crescentus* (Christen et al., 2010). Fluorescent probes utilizing the PilZ protein MrkH, c-di-GMP-dependent dimerization of BldD, and a BRET probe fusing a YFP, Venus, and Split RLuc to YcgR have also been developed to visualize c-di-GMP in bacterial cells (Ho et al., 2013; Dippel et al., 2018; Halte et al., 2022). In this study, we have shown that the c-di-GMP probe YFP-YcgR-CFP also works in *Dictyostelium* cells to allow live imaging of c-di-GMP signaling in multicellular stages.

## 2 Materials and methods

### 2.1 Cell strains and culture conditions

*D. discoideum* strains used in this study are listed in Supplementary Table S1. Cells were grown axenically in HL5 medium including glucose (Formedium, United Kingdom) in 90-mm culture dishes at 21°C. Cells for fluorescence imaging were grown in low-fluorescence axenic medium containing 1 mL 1000 × FM salts 1, 1 mL 1000 × FM salts 2, 0.1 mL 10000 × FM trace elements, 11 g glucose·1H_2_O, 5 mL 1 M K_2_HPO_4_ and 5 g casein peptone per liter, and the pH was adjusted to 6.5 as described in DictyBase (http://dictybase.org/). 1000 × FM salts 1 contains 500 mM NH_4_Cl, 200 mM MgCl_2_ and 10 mM CaCl_2_. 1000 × FM salts 2 is 50 mM FeCl_3_. 10000 × FM trace elements is 100 mM Na_2_-EDTA·2H_2_O, 130 mM ZnSO_4_·H_2_O, 140 mM H_3_BO_4_, 25 mM MnCl_2_·4H_2_O, 7 mM CoCl_2_·6H_2_O, 6 mM CuSO_4_·5H_2_O, and 0.18 mM (NH_4_)6Mo7O_24_·4H_2_O. Transformants were maintained in medium containing 20 μg/mL G418 (Fujifilm Wako, Japan).

### 2.2 Plasmid construction and genetic manipulation

Plasmids used in this study are listed in Supplementary Table S2. pDM304/mYpet-YcgR-mCypet was constructed by insertion of a mYpet-YcgR-mCypet fragment from addgene plasmid #90102 into the *Bgl*II and *Spe*I sites of pDM304 (Veltman et al., 2009). The R118A point mutation was introduced to pDM304/mYpet-YcgR-mCypet plasmid by site-directed mutagenesis method (Kunkel, 1985) using DNA polymerase (KOD One, TOYOBO) and primers. The wild-type AX2 strain was transformed with ∼1.5 μg plasmid using a electroporator (MicroPulser, Bio-Rad); transformants were selected with 20 μg/mL G418.

### 2.3 Fluorescence spectrophotometry

Cells expressing the FRET sensor were starved in developmental buffer (DB: 5 mM Na_2_HPO_4_, 5 mM KH_2_PO_4_, 2 mM MgCl_2_, 0.2 mM CaCl_2_, pH 6.5) at a density of 1.0 × 10^7^/mL for 3 h and lysed in ice cold lysis buffer containing 10 mM Tris-HCl, pH7.5, 0.2 mM EGTA, 200 mM sucrose as described by Bagorda et al. (Bagorda et al., 2009). The fluorescence intensities with or without 0.1, 0.5, 1.0, or 10 µM c-di-GMP (SML1228, Sigma-Aldrich) were measured with excitation 430 nm and emission 460 and 520 nm using fluorescence spectrophotometer (FP-8550, Jasco). The ratio of CFP/FRET values were calculated the emission value at 460 nm divided by the emission value at 520 nm.

### 2.4 Image acquisition and analysis

In all experiments, cells were observed at 21°C. Microscopic images were taken using an inverted microscope (IX83, Evident) equipped with an sCMOS camera (Prime 95B, Photometrics) and objectives (UPLSAPO 20X/0.75 NA or UPLXAPO 40X/0.95 NA, Evident). The c-di-GMP sensor was excited by a 130 W mercury light source system (U-HGLGPS, Evident) with a fluorescence mirror unit CFP-2432C-OFF (Excitation BP 438/24; Emission BP 483/32, Semrock) for CFP images and a customized mirror unit (Excitation BP 438/24; Emission BP 542/27, Semrock) for FRET images. All images were processed and analyzed using Fiji (Schindelin et al., 2012). Background intensity was defined as the mean pixel intensity of the ROI without cells. For ratiometric imaging, the fluorescence intensity of the CFP image was divided by the intensity of the FRET image using the CFP and FRET images subtracted the background intensity, respectively.

### 2.5 FRET imaging

To induce development, cells at the exponential phase (1.5–3 × 10^6^/mL) were harvested and washed three times in reverse osmosis (RO) water. Multicellular bodies were allowed to form on agar plates and observed as described (Hashimura et al., 2019). Multicellular bodies were imaged using inverted fluorescence microscopy.

## 3 Results

### 3.1 Expression of the c-di-GMP fluorescent sensor

**FIGURE 1:**
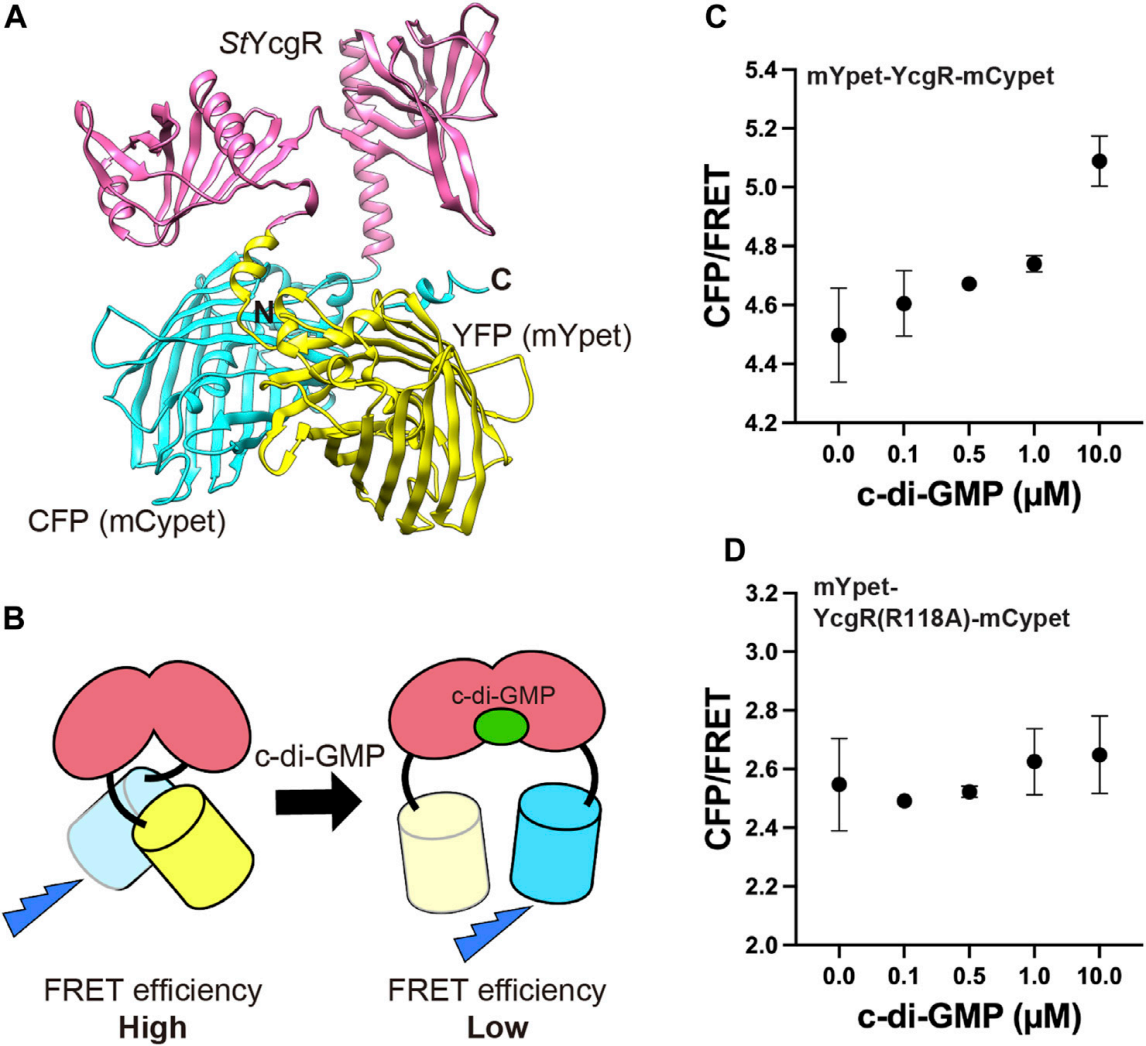
FRET c-di-GMP sensor. **(A)** Structure of mYpet-YcgR-mCypet (Christen et al., 2010) predicted by AlphaFold2. The sensor is a fusion of mYpet (yellow), *Salmonella* YcgR (magenta) and mCypet (cyan). **(B)** c-di-GMP sensor have decreased FRET efficiency when c-di-GMP binds to the YcgR part. FRET efficiencies of the mYpet-YcgR-mCypet **(C)** and mYpet-YcgR(R118A)-mCypet **(D)** from *Dictyostelium* cell lysates with or without 0.1, 0.5, 1.0, or 10 µM c-di-GMP at 23°C, respectively. CFP/FRET values were calculated CFP intensity divided by FRET intensity. Measurements were taken four times for each concentration. Error bars show standard deviations.

The FRET-based c-di-GMP sensor utilized in bacteria (Christen et al., 2010) was expressed under the constitutive act15 promoter in *D. discoideum* AX2. It consists of mYpet and mCypet fused to the N- and C-termini of *Salmonella* YcgR, respectively. Prediction of the sensor protein structure was performed using a machine-learning model AlphaFold2 (Jumper et al., 2021) (Figure 1A). Since the FRET efficiency of the sensor decreases when bound to c-di-GMP, an increase in c-di-GMP concentration decreases FRET efficiency (Figure 1B). To confirm that the FRET sensor is functional, cells expressing it were lysed and the dependence of the FRET efficiency on c-di-GMP concentration for the sensor produced in *Dictyostelium* cells was examined. Changes in fluorescence intensity with or without 0.1, 0.5, 1.0 or 10 µM c-di-GMP were measured using spectrophotometry. Increasing concentrations of c-di-GMP added to the lysate decreased FRET efficiency and increased the CFP/FRET ratio (*p* < 0.001, one-way ANOVA), confirming its utility as a sensor (Figure 1C). Since the 118th arginine residue of YcgR is conserved and known to be critical for c-di-GMP binding (Ryjenkov et al., 2006; Hou et al., 2020), the single-residue substitution R118A was made to YcgR, yielding mYpet-YcgR(R118A)-mCypet, and the lysate of cells expressing it were tested, but no significant FRET changes for c-di-GMP were observed (one-way ANOVA) (Figure 1D). Considering that *Dictyostelium* responds to more than 1 µM external c-di-GMP stimulation for gene expression (Chen et al., 2017) and that c-di-GMP functions at concentrations ranging from tens of nM to tens of µM in bacterial cells (Christen et al., 2010; Pultz et al., 2012) that utilize c-di-GMP, this sensor is suitable for intracellular c-di-GMP monitoring.

### 3.2 Fluorescent imaging of c-di-GMP in multicellular bodies

**FIGURE 2:**
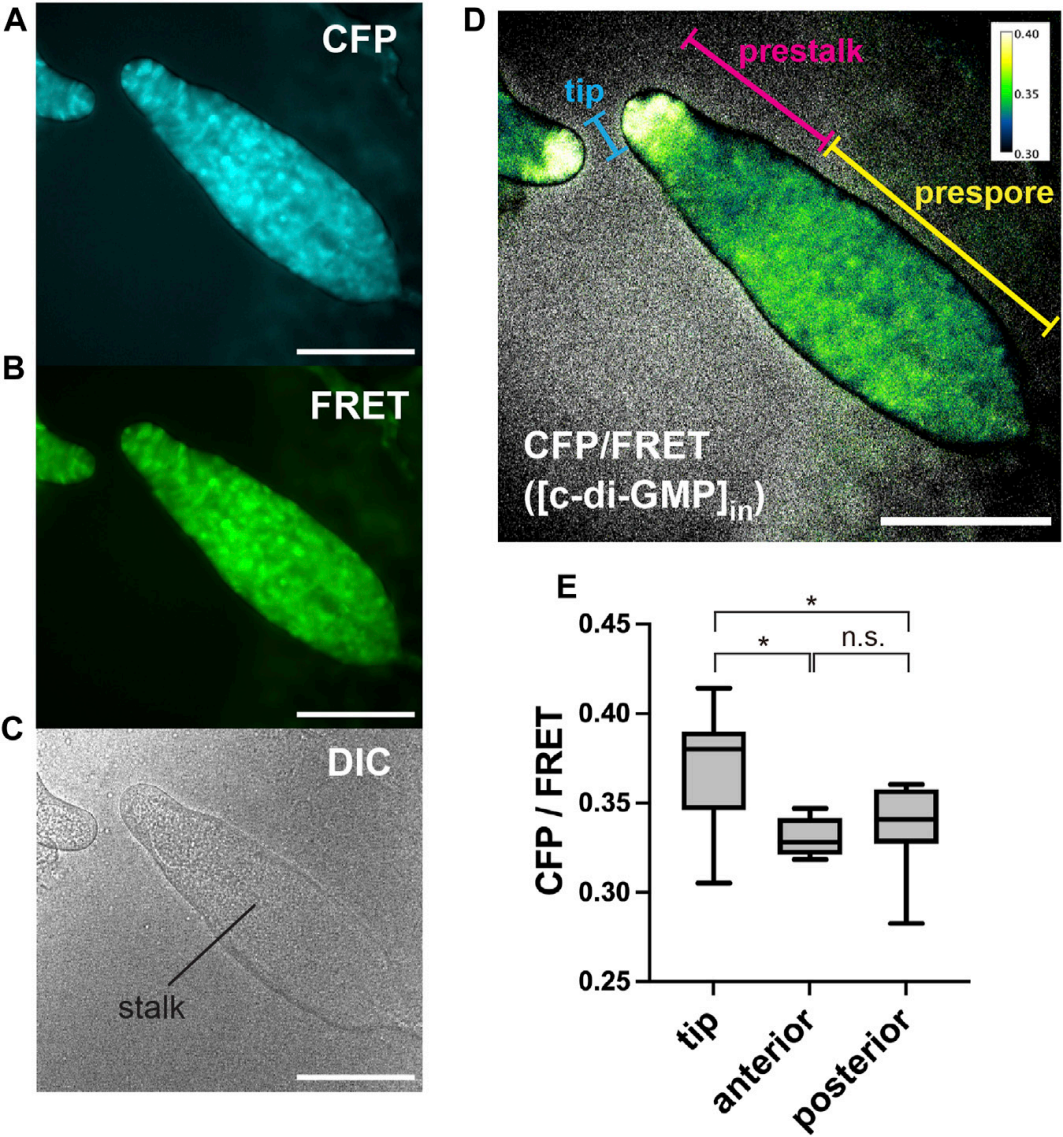
Representative images of c-di-GMP in *Dictyostelium* multicellular bodies of CFP **(A)**, FRET **(B),** and DIC **(C)**. **(D)** Ratiometric FRET image created by dividing the CFP image by the FRET image. Ratios are shown in pseudocolor (inset). Scale bars, 100 µm. **(E)** CFP/FRET ratios for multicellular body regions (*n* = 9). Anterior is defined as the prestalk region minus the tip region. Posterior is the prespore region. *p < 0.05 (ANOVA with Tukey’s multiple comparisons test); n.s., no significant difference.

The fluorescence of c-di-GMP in single cells could be measured, but its brightness was not high; moreover, the FRET changes were not very large (Figure 1C). To better capture subtle changes in intracellular FRET, it was necessary to suppress autofluorescence. Utilizing a medium that has been shown to reduce autofluorescence described in DictyBase (Basu et al., 2015), the autofluorescence of multicellular bodies could be reduced at the emission wavelength (Supplementary Figure S1). Cells expressing the c-di-GMP sensor were cultured in this low-fluorescence medium and then starved to initiate development. In culminant stages, the c-di-GMP signal was observed to be greater in the tip region (Figure 2), where the boundary between the prestalk and prespore regions can be distinguished by a neck (Bonner, 1998; Gaudet et al., 2008). However, we observed no difference in signal between the prespore region and the prestalk regions, except for the tip (Figure 2E). This high concentration in the tip is not consistent with the reported distribution of its synthetic enzyme DgcA (Chen and Schaap, 2012), but is consistent with the distribution of the induced adenylyl cyclase AcaA, thought to be activated by c-di-GMP (Chen et al., 2017). Since the *K*_*d*_ of the c-di-GMP sensor is 195 nM (Christen et al., 2010), we infer that the tip area has a concentration of at least several hundred nM.

### 3.3 Time-lapse measurement of c-di-GMP during development

**FIGURE 3:**
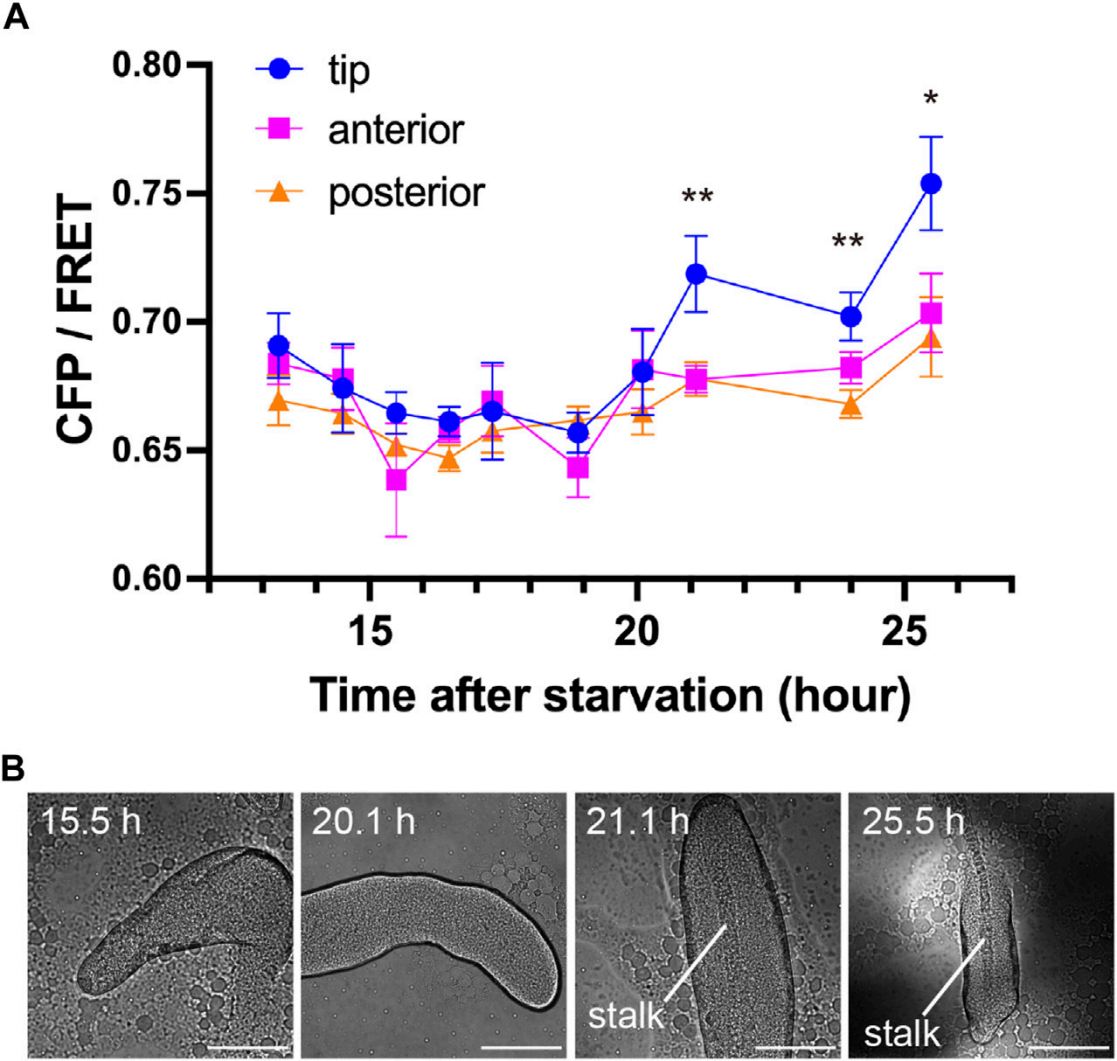
Transition of c-di-GMP synthesis during development. **(A)** CFP/FRET ratios for each region within the multicellular body at each time point. Means ± standard errors are shown (*n* = 19); *p < 0.05; **p < 0.01 (ANOVA with Tukey’s multiple comparisons test). The absence of asterisks indicates no significant difference. **(B)** Representative DIC micrographs of *Dictyostelium* multicellular bodies at four time points. Scale bars, 100 µm.

**FIGURE 4:**
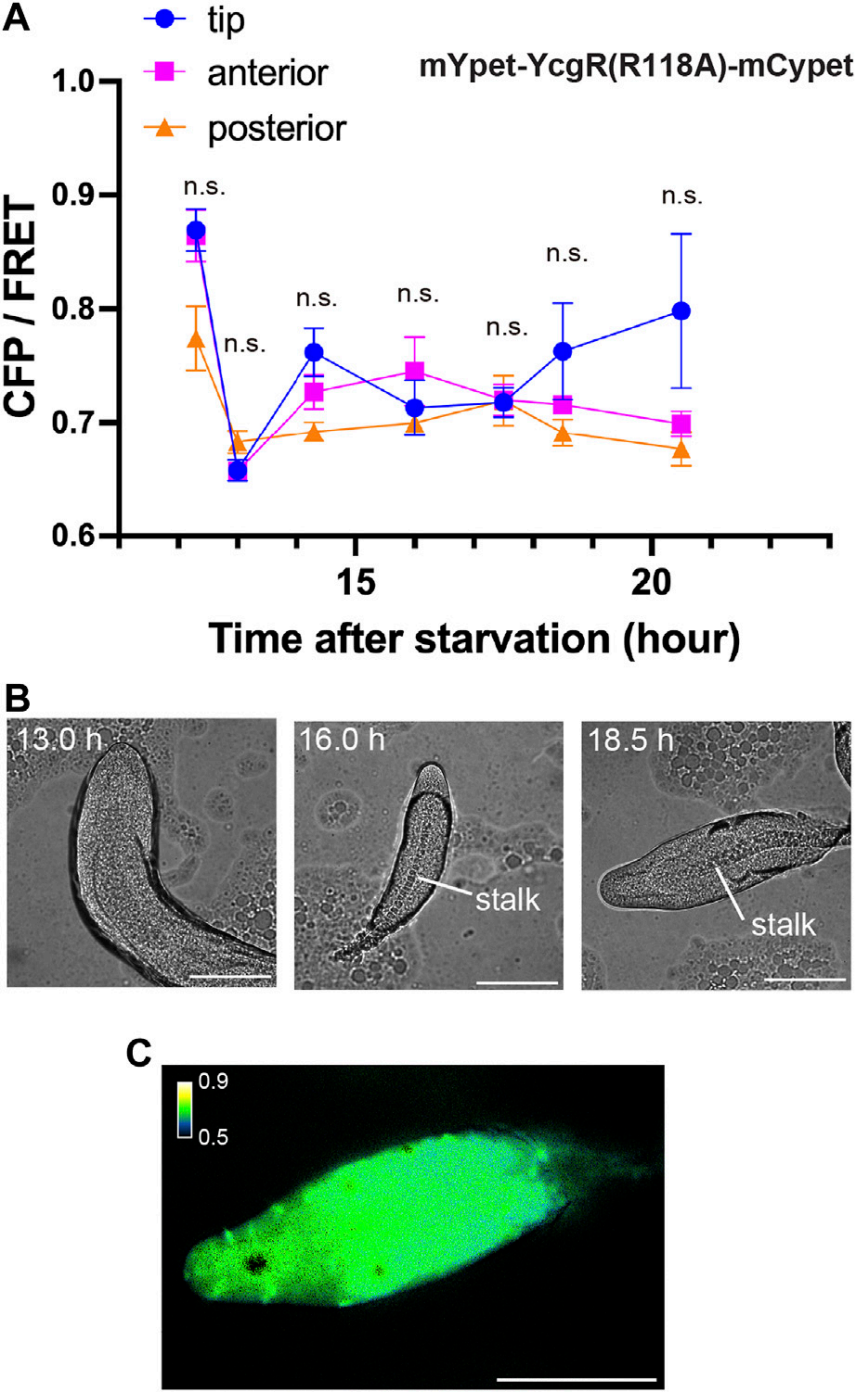
Measurement of mutant mYpet-YcgR(R118A)-mCypet during development. **(A)** CFP/FRET ratios of mYpet-YcgR(R118A)-mCypet for regions of the multicellular body at each time point. Means ± standard deviations are shown (*n* = 19). Data were assessed using ANOVA with Tukey’s multiple comparisons test; n.s., no significant difference. **(B)** Representative DIC micrographs of *Dictyostelium* multicellular bodies at three time points in development. **(C)** A representative ratiometric FRET image of the multicellular body at 18.5 h. Scale bars, 100 µm.

To determine at what point during development the concentration of c-di-GMP in the tip begins, its signal was monitored during development from initial multicellularity (13 h after starvation) to the formation of fruiting bodies (Figure 3). Immediately after slug formation, there was no clear localization of c-di-GMP, but a strong signal at the tip was observed when stalk formation was confirmed inside the multicellular body (Figures 3A, B). Figure 2, showing c-di-GMP localization, also shows stalk formation (Figure 2C). Although cellular autofluorescence increases with fruiting body formation, we observed no significant difference between the tip and other regions in AX2 multicellular bodies not expressing fluorescent sensors (Supplementary Figure S2). The mYpet-YcgR(R118A)-mCypet does not respond significantly to c-di-GMP (Figure 1D). Time-lapse measurements of the development of cells expressing this probe showed no significant change in FRET efficiency (Figure 4A). The faint but non-significant difference in the tip region after 18.5 h may be due to the weak affinity of the YcgR(R118A) mutant, which has been reported to be *K*_*d*_ of 14 µM (Hou et al., 2020) (Figure 4). These results suggest that a large amount of c-di-GMP is synthesized during stalk formation at the tip of the culminant.

### 3.4 c-di-GMP in the *dgcA* mutant

**FIGURE 5:**
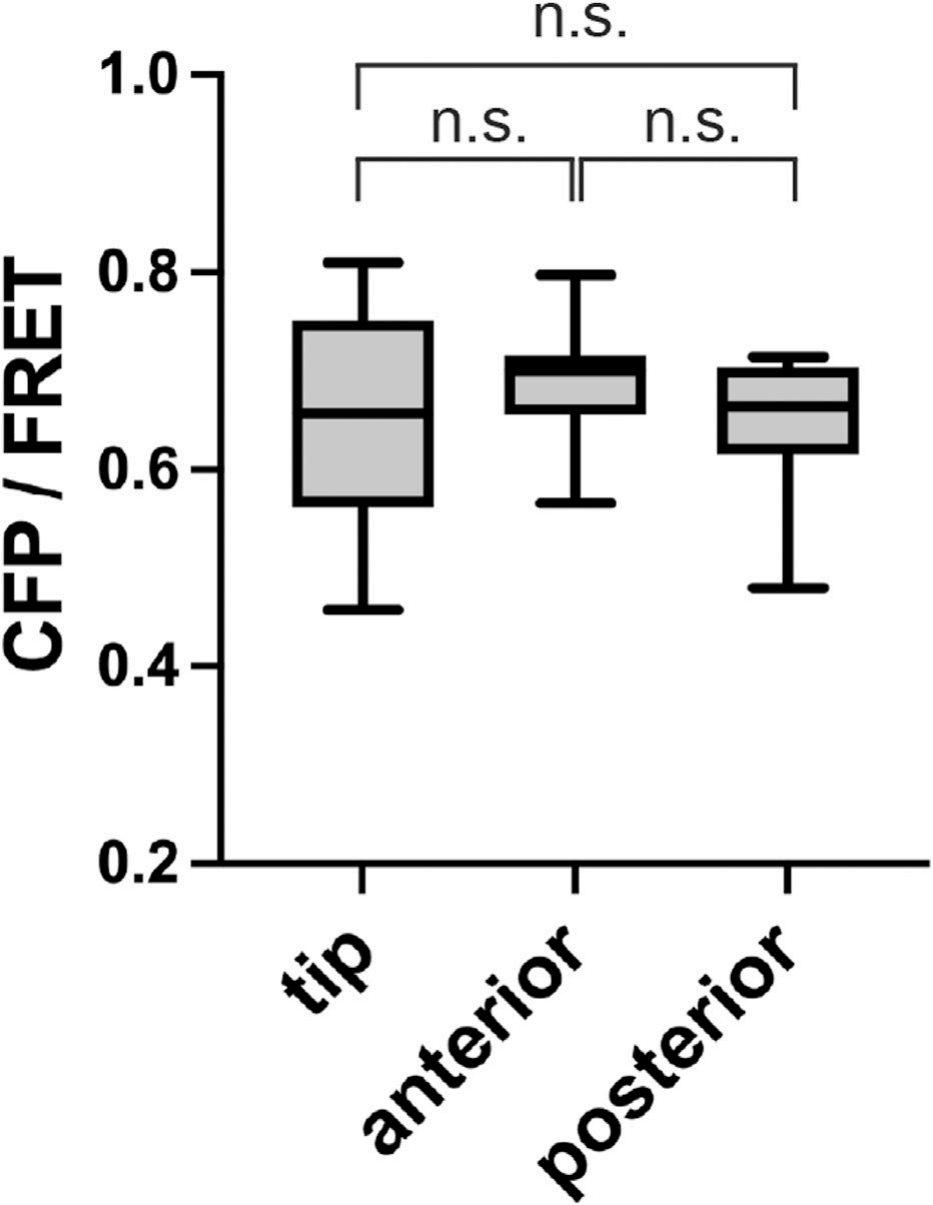
No localization of c-di-GMP in a *dgcA* mutant. CFP/FRET ratios for each slug region are shown as box plots (*n* = 10). Anterior is defined as the prestalk region minus the tip region. Posterior is the prespore region. Data were assessed with ANOVA with Tukey’s multiple comparisons test; n.s., no significant difference.

*D. discoideum*, DgcA synthase is primarily responsible for the synthesis of c-di-GMP (Chen and Schaap, 2012; Song et al., 2015; Chen et al., 2017). The *dgcA* mutant does not form fruiting bodies because c-di-GMP does not induce stalk-cell differentiation, but they do progress to the slug stage (Chen and Schaap, 2012). FRET in the *dgcA* mutant was performed after multicellular body formation, with no significant difference between the regions (Figure 5). This indicates that the wild-type c-di-GMP signal (Figures 2D, 3A) depends on the c-di-GMP synthesized by DgcA synthase.

## 4 Discussion

In *D. discoideum*, stalk cell differentiation is induced by c-di-GMP and DIF-1 (Morris et al., 1987; Thompson and Kay, 2000; Saito et al., 2008; Chen and Schaap, 2012; Kin and Schaap, 2021). Since this differentiation also induces cell death, it provides information on cell-death mechanisms (Song et al., 2015). The presence of c-di-GMP activates AcaA in tip cells and cAMP synthesized by AcaA in turn activates PKA to induce the differentiation of prestalk cells into stalk (Chen et al., 2017). Conversely, ammonia acts on RegA via DhkC, which suppresses cAMP signaling, thereby inhibiting stalk differentiation (Schindler and Sussman, 1977; Singleton et al., 1998). Our observation of c-di-GMP accumulation in the tip during stalk formation (Figures 2, 3) is consistent with the localization of AcaA (Verkerke-van Wijk et al., 2001), supporting the previous spatiotemporal model (Chen et al., 2017). On the other hand, the fact that the distribution of the synthetic enzyme DgcA did not match the distribution of c-di-GMP may indicate that DgcA is activated only in tip cells, or that c-di-GMP synthesis is suppressed in prestalk cells except for the tip. The activity of some bacterial diguanylates are known to be regulated by partner proteins (Chen et al., 2021). There are proteins that are expressed only at the tip or prestalk region of *Dictyostelium* multicellular bodies (Williams, 2006); it is possible that they include regulatory partners for DgcA activity. Alternatively, since acidification is known to act on stalk differentiation (Gross et al., 1983), differences in the cellular environment, such as intracellular pH, may regulate DgcA activity spatially and temporally.

Cells not expressing the sensor, or expressing a probe that did not bind c-di-GMP, had normal stalk development at 16 h or more after starvation; however, in cells expressing the c-di-GMP sensor, stalk formation was delayed to 21 h or more after starvation (Figures 3, 4). This is likely due to the binding of c-di-GMP to the sensor reducing intracellular free c-di-GMP. This is also an indication that c-di-GMP is important for stalk formation.

This study visualized c-di-GMP signaling in eukaryotes for the first time (Figure 2). To elucidate more of the spatiotemporal dynamics of c-di-GMP at the single-cell or single-local levels, it is necessary to increase sensor sensitivity. The *K*_*d*_ of the sensor used in this study is approximately 195 nM (Christen et al., 2010), making it suitable for *Dictyostelium* because c-di-GMP stimulations above 1 µM induce expression of stalk-specific genes (Chen et al., 2017); however, we only observed significant differences during stalk-cell formation, which is considered to have the highest intracellular c-di-GMP concentration. DgcA is widely expressed in prestalk cells and may function at lower concentrations; alternatively, it may be produced at an earlier time point. PilZ domain protein PA3353 from *Pseudomonas aeruginosa* has two binding sites, one of which with a *K*_*d*_ of 88 nM, and may be used as a sensitive c-di-GMP sensor (Pultz et al., 2012). As development progresses to multicellular and fruiting bodies, autofluorescence increases, which affects the fluorescence ratio imaging (Figure 4A; Figure 5). The BRET sensor may be useful for measurement with high signal-to-noise ratio, because it can greatly decrease artifacts caused by autofluorescence (Dippel et al., 2018). The c-di-GMP sensor used in this study was not bright enough and required intense excitation, making it difficult to measure at the single-cell level. Since c-di-GMP acts as an extracellular signal (Chen and Schaap, 2016; Chen et al., 2017), measurement of signal dynamics upon stimulation is important. Recently, multiple stable fluorescent proteins have been developed (Hirano et al., 2022), and their use in sensors may enable measurement of responses to external c-di-GMP stimulation.

*D. discoideum* synthesize and utilize c-di-GMP signal by themselves using lateral gene transfer from bacteria (Chen and Schaap, 2012; Kin and Schaap, 2021). Therefore, *D. discoideum* is the only known eukaryote species that uses c-di-GMP as a signal (Chen and Schaap, 2012). In mammalian cells, c-di-GMP is recognized as a bacterial signal by the STING pathway and innate immunity functions (Burdette et al., 2011; Yin et al., 2012). The use of *Dictyostelium* cells as a model for measuring c-di-GMP signaling in eukaryotic cells would provide a model for immunological studies.

## Data Availability

The original contributions presented in the study are included in the article/Supplementary Materials, further inquiries can be directed to the corresponding author.
