# Supplementary material for: Visualization of c-di-GMP in multicellular Dictyostelium stages
Source: Front Cell Dev Biol. 2023 Jul 20;11:1237778. doi: 10.3389/fcell.2023.1237778 (PMC10399225; doi:10.3389/fcell.2023.1237778)
Supplement: Supplementary file 1 [file DataSheet1.pdf]

Supplementary Materials

Visualization of c-di-GMP signal in *Dictyostelium* multicellular body

Hayato Ide, Yukihisa Hayashida, Yusuke V. Morimoto*

*** Correspondence:** Yusuke V. Morimoto: yvm001@phys.kyutech.ac.jp

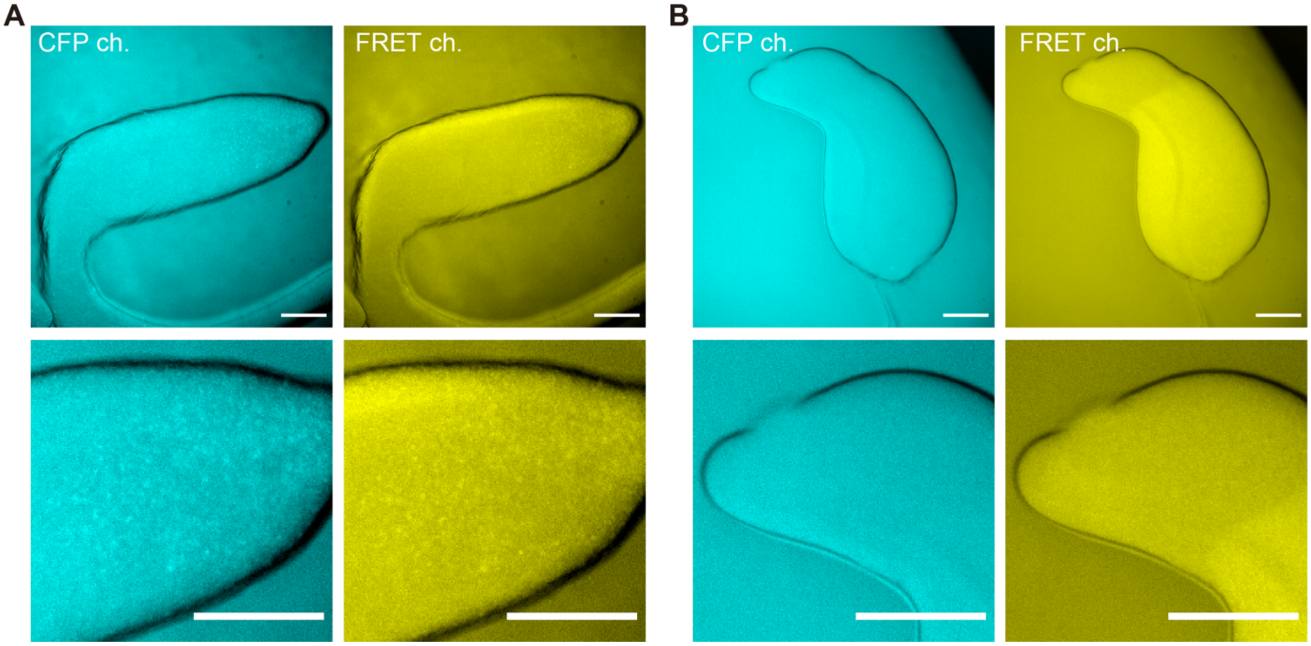

Supplementary Figure S1. Representative fluorescence images of autofluorescence in *Dictyostelium* multicellular body are shown. **(A)** Fluorescence images of autofluorescence of a multicellular body grown in HL5 medium including glucose. The lower panels show the enlargement of upper images. **(B)** Fluorescence images of autofluorescence of a multicellular body grown in low fluorescence medium. The lower panels show the enlargement of upper images. Scale bars, 100 µm.

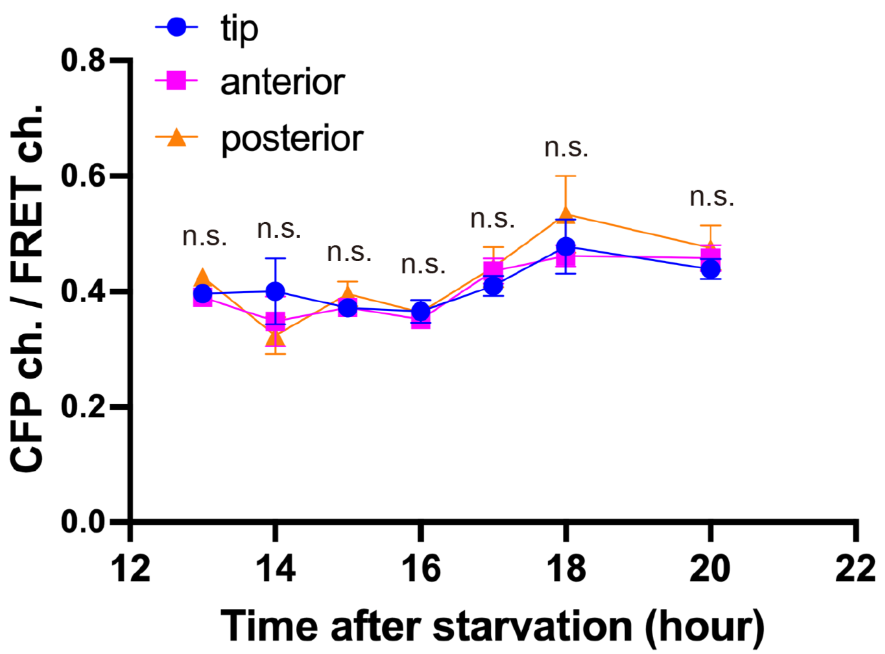

Supplementary Figure S2. Timelapse measurement of the autofluorescence in AX2 during development. Ratio values of CFP/FRET channels for each region within the multicellular body at each time point after starvation. Mean ± standard errors are shown (n = 9). Data were assessed with ANOVA with Tukey's multiple comparisons test; n.s., no significant difference.

Supplementary Table S1

Dictyostelium discoideum strains used in this study.

**Strain name**	**Characteristics**	**Background**	**Source or reference**
AX2	wild type	AX2	Lab stock
dgcA^{-}	*dgcA*$^{-}$ (bsR)	AX2	(Chen and Schaap, 2012)<br>NBRP-nenkin (ID: S90402)
mYpet-YcgR-mCypet /AX2	(neoR)	AX2	This study
mYpet-YcgR(R118A)-mCypet /AX2	(neoR)	AX2	This study
mYpet-YcgR-mCypet /*dgcA*$^{-}$	(neoR)	AX2	This study

Supplementary Table S2

Plasmids used in this study.

**Plasmid name**	**Characteristics**	**Backbone**	**Source or reference**
pMMB67EH-Spy	mYpet-YcgR-mCypet	pMMB67EH	(Christen et al., 2010)<br>Addgene (#90102)
pDM304/mYpet-YcgR-mCypet	mYpet-YcgR-mCypet	pDM304 (neoR)	This study
pDM304/mYpet-YcgR(R118A)-mCypet	mYpet-YcgR(R118A)-mCypet	pDM304 (neoR)	This study
